# Clinical features and outcomes of orthopaedic injuries after the kahramanmaraş earthquake: a retrospective study from a hospital located in the affected region

**DOI:** 10.1186/s13049-024-01181-6

**Published:** 2024-01-30

**Authors:** Murat Gök, Mehmet Ali Melik

**Affiliations:** Medicalpoint Gaziantep Hospital, Mücahitler 52063. Sk. No: 2,27584 Şehitkamil, 27100 Gaziantep, Turkey

**Keywords:** Earthquake, Compartment syndrome, Trauma, Crush injury, Fasciotomy

## Abstract

**Background:**

The purpose of this retrospective, single-institutional study was to report the clinical features and outcomes of orthopaedic injuries after the Kahramanmaraş earthquake.

**Methods:**

An institutional database review was conducted to evaluate the results of patients who applied to our hospital’s emergency department after the Kahramanmaraş earthquake. Trauma patients referred to orthopaedics and traumatology were identified. Patient records were checked for injury type, fracture site, treatment type (conservative or surgical), surgical technique, and outcome. Diagnosis with crush syndrome and the need for haemodialysis were also noted. Bedside fasciotomy was undertaken based on the urgency of the patient’s condition, number of patients and the availability of the operating theatre. A team consisting of a trauma surgeon, a plastic surgeon, a board-certified physician in infectious disease, a reanimation specialist, a general surgeon and a nephrologist followed up with the patients.

**Results:**

Within the first 7 days following the earthquake, 265 patients were admitted to the emergency department, and 112 (42.2%) of them were referred to orthopaedics and traumatology. There were 32 (28.5%) patients diagnosed with acute compartment syndrome. Fasciotomy was performed on 43 extremities of 32 patients. Of these extremities, 5 (11.6%) were upper and 38 (88.4%) were lower extremities.The surgeries of 16 (50%) of the patients who underwent fasciotomy were performed in the emergency department. There was no significant difference in terms of complications and outcomes between performing the fasciotomy at the bedside or in the operating theatre (*p* = 0.456).

**Conclusions:**

Fasciotomy appears to be a crucial surgical procedure for the care of earthquake causalities. Fasciotomy can be safely performed as a bedside procedure based on the urgency of the patient’s condition as well as the availability of the operating theatre.

## Introduction

Earthquakes are natural disasters that cannot be accurately predicted in terms of location or time before they occur. On average, approximately 15 destructive earthquakes with a magnitude of over 7 occur each year worldwide [1].

On February 6th, 2023, at 04:17 local time, a devastating earthquake with a magnitude of 7.8 occurred in Kahramanmaraş, Turkey (37.166°N 37.042°E) [2]. Forty-two hospitals in the region suffered moderate to severe damage [3]. Additionally, 448 health care workers lost their lives because of the earthquake [4]. At the time of this study, there were 50,096 deaths, and 107,204 people were injured [5].

Extremity trauma is the most common type of injury observed in individuals who are rescued alive from under debris after an earthquake [6, 7]. Complications, such as bleeding, infection, sepsis, and crush syndrome, can result in the loss of rescued survivors [8]. A multidisciplinary approach is required for the treatment of the victims [9].

The purpose of this retrospective, single-centre study was to report the clinical features and outcomes of orthopaedic injuries after the Kahramanmaraş earthquake.

## Methods

This retrospective study was carried out with approval from the local ethics committee (approval number: 2023/70). An institutional database review was conducted to determine individual causalities among patients admitted to the emergency department of our hospital after the Kahramanmaraş earthquake. The hospital where the study was conducted is a secondary care facility with a capacity of 265 inpatient beds and 89 intensive care unit beds. Trauma patients referred to orthopaedics and traumatology were identified. Patients whose medical records were not sufficient, who were transferred to another center during treatment, or whose follow-up could not be completed were excluded from the study. Medical records of patients admitted in the first 7 days were checked for age, gender, admission time, injury type, fracture site, treatment type (conservative or surgical), surgical technique, and outcome. Diagnosis with crush syndrome and the need for haemodialysis were also noted.

**Fig. 1:**
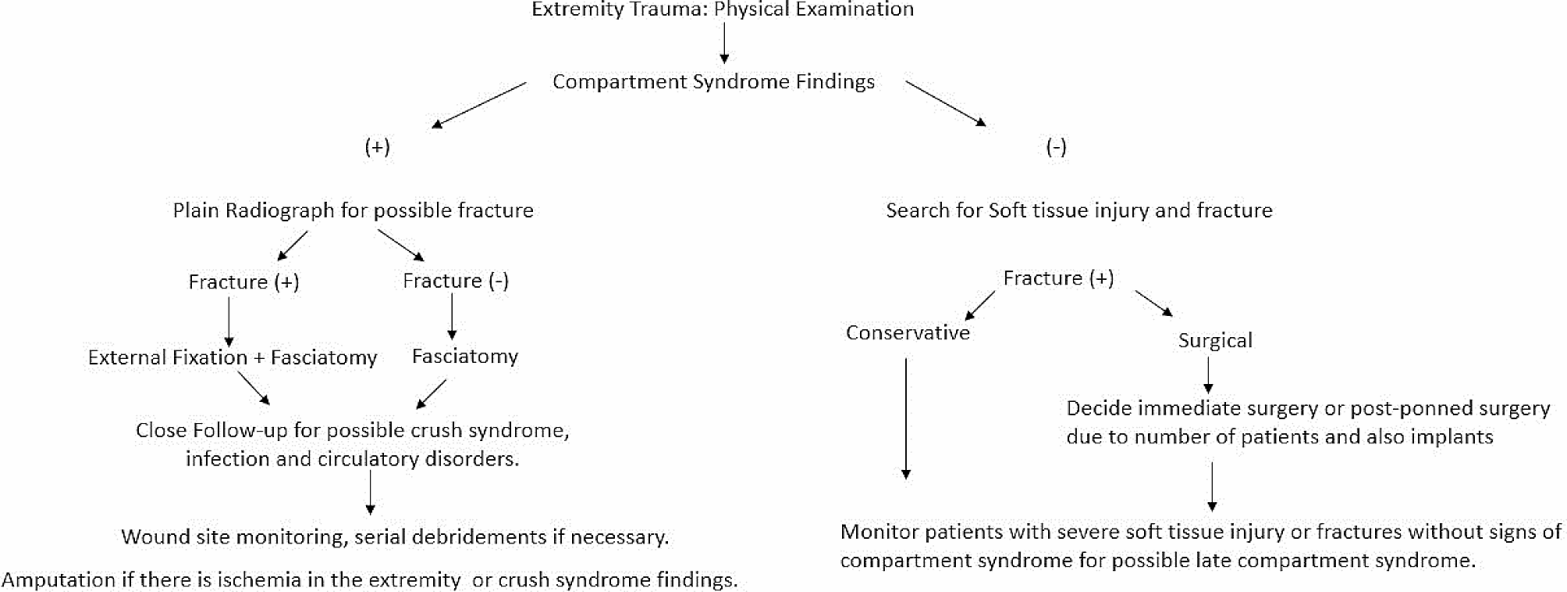
The protocol we applied to patients with extremity injuries after the earthquake

A urinary catheter was placed in all patients rescued from the wreckage and/or with crush injuries to their extremities. Hourly Urine output and urine colour were monitored. Baseline blood samples were collected and complete blood count, urea, creatinine, bleeding time, creatine kinase, aspartate aminotrensferase, alanine aminotransferase, sodium, potassium values were measured. After physical examination, imaging procedures were applied for patients with indication. The diagnosis of acute compartment syndrome was made based on clinical signs and symptoms. Patients were examined for typical findings of acute compartment syndrome, including spontaneous pain, pain with passive extension, paralysis, paraesthesia, and the presence of oedema [10]. However, intracompartmental pressure measurement was not performed for any of the patients. Plain radiography was performed for all extremities diagnosed with compartment syndrome (Fig. 1).

The diagnosis of crush syndrome was made in patients with crush injuries by monitoring increased creatine kinase levels, renal function, potassium levels and urine output. A diagnosis of fracture was made based on clinical and plain radiographic findings.

All surgical procedures were performed by two certified trauma surgeons. Bedside fasciotomy was undertaken based on the urgency of the patient’s condition, number of patients and as well as the availability of the operating theatre. However, the individual surgeon decided whether to perform fasciotomy as a bedside procedure or in the operating theatre. Bedside fasciotomies were performed in the emergency department by the surgical team. Regarding anaesthesia, all patients received 2 mg/kg propofol (Diprivan®) whether the procedure was performed at bedside or in the operating theatre. Before the procedure, two grams of cefazolin was administered intravenously for prophylaxis. A tourniquet was not used. Lower extremity fasciotomies were performed by releasing four compartments using a single incision on the lateral side [11]. For the upper extremity, an incision was created starting from the palmar side, passing through the wrist in an inclined manner, and extending proximally to the elbow. The lacertus fibrosus was released in all upper extremity fasciotomies [12]. No approximated sutures were used after the surgery, and wound care was provided with fusidic acid ointment. Soft tissue infection, excessive bleeding after surgery, and sepsis were recorded as surgical site-related complications. The diagnosis of soft tissue infection was made based on clinical findings. For fractures, surgical treatment, plate screws, temporary external fixators, intramedullary nails, k-wires, and titanium elastic nails were used according to the requirements of the patients. Fractures in patients who were deemed suitable for conservative treatment or for whom surgery could be postponed were immobilized with casts. In the conservative treatment of fractures, devices such as casts, splints and slings were used. Skin laceration, abrasion, muscle damage, tendon and ligament injuries are considered soft tissue injuries. In the treatment of soft tissue injuries; surgically repaired or conservatively; compressive bandages, splints, dressings, and non-steroidal anti-inflammatory drugs were used.

Fasciotomies performed within the first 24 h after trauma were considered early, and those performed after 24 h were considered late. After fasciotomy, a team consisting of a trauma surgeon, a plastic surgeon, a board-certified physician in infectious disease, a reanimation specialist, a general surgeon and a nephrologist followed up with the patients. The outcome of the patients were noted after a follow-up of two months.

We employed SPSS version 20.0 statistical software (IBM Corp., Armonk, NY, USA) for statistical analyses. Categorical variables are reported as numbers and percentages, whereas continuous variables are reported as medians and ranges. The Kolmogorov‒Smirnov test was used to examine the normality of the distribution of numerical variables. The chi-square test and Fisher’s exact test were used to compare categorical variables, and the Mann–Whitney and Kruskal–Wallis tests were used for comparing intergroup distributions of continuous variables. For all analyses, *p* < 0.05 was considered statistically significant.

## Results

**Fig. 2:**
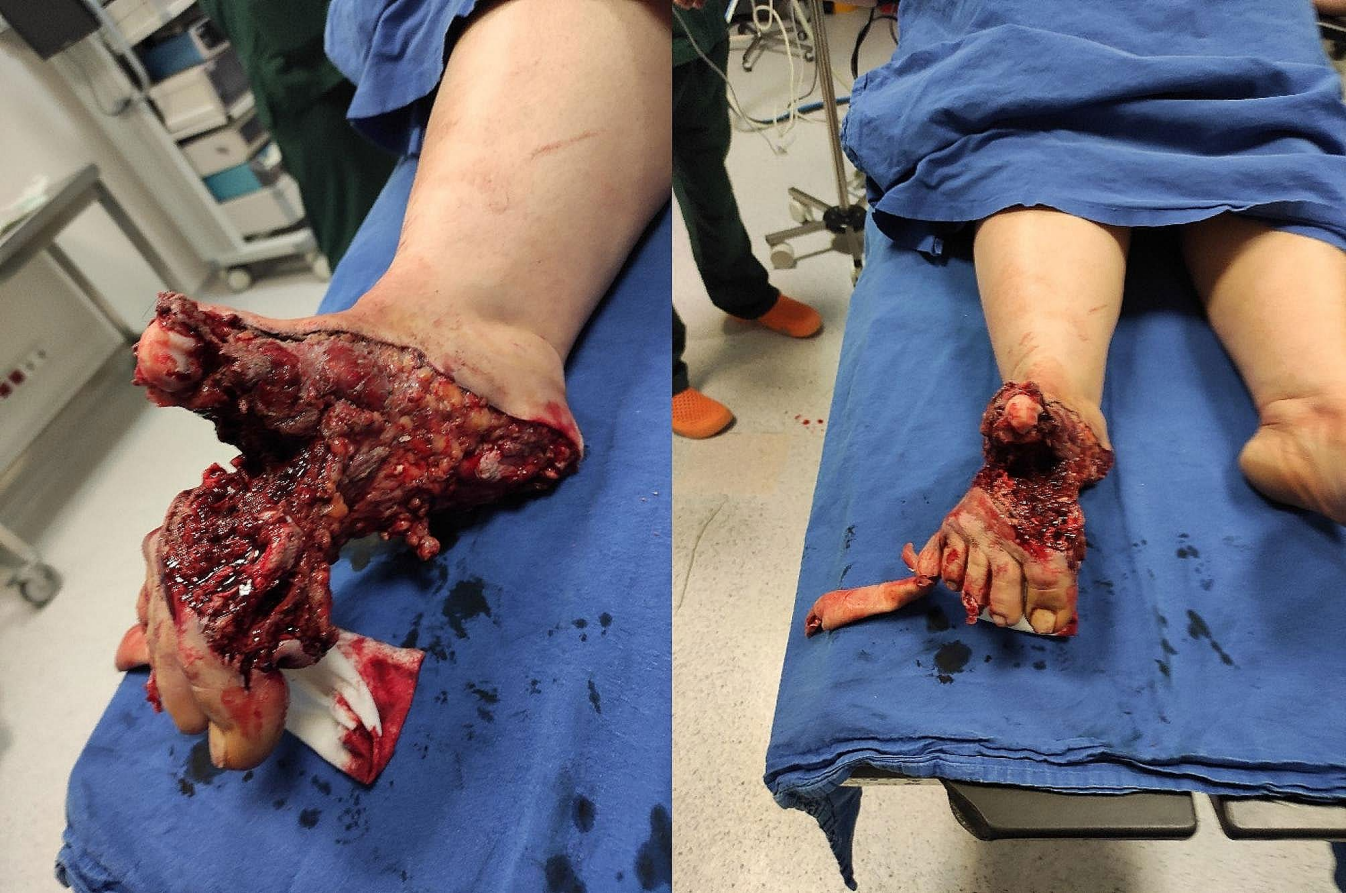
Traumatic amputation of foot

Within the first 7 days following the earthquake, 265 patients were admitted to the emergency department, and 112 (42.2%) of them were referred to orthopaedics and traumatology. The median age of orthopaedic patients was 32.0 years (range, 3–89 years). Fifty-eight (51.8%) of the patients were female, whereas 54 (48.2%) were male. Most of the admissions were on the first (*n* = 41 [36.6%]) and second day (*n* = 33 [29.4%]) following the earthquake. Sixty-eight (60.7%) of the orthopaedic patients underwent surgery, and 44 (39.3%) underwent conservative treatment (Table 1). Out of 91 surgeries performed at our hospital in the first seven days following the earthquake, 68 (74.7%) were carried out by the orthopaedics and traumatology team. There were 32 (28.5%) patients diagnosed with acute compartment syndrome. However, 22 (19.6%) patients had lower extremity fractures, 20 (17.8%) had upper extremity fractures, one had an elbow dislocation (0.8%), five (4.4%) had pelvic fractures, 29 (25.8%) had soft tissue injuries, and three (2.6%) patients underwent amputation due to severe trauma (Fig. 2) (Table 2).

**Table 1:** Demographic characteristics of the orthopaedic patients

Variable	Number of Patients, n = 112
Sex; n (%)	
Female	58 (51.8%)
Male	54 (48.2%)
Age; Median (Range)	32 (3–89)
Age; Mean ± SD	
Female	37.53 ± 20.27
Male	30.39 ± 15.89
Treatment; n (%)	
Surgical	68 (60.7%)
Conservative	44 (39.3%)

**Table 2:** Injury types of orthopaedic patients

Injury Type	Upper Extremity n (%)	Lower Extremity n (%)	Total n (%)
Fracture*	20 (17.8%)	27** (24.1%)	47 (41.9%)
Dislocation	1 (0,8%)	-	1 (0.8%)
Acute Compartment Syndrome*	5 (4.5%)	27 (24.1%)	32 (29.6%)
Traumatic Amputation	2 (1.8%)	1 (0.9%)	3 (2.7%)
Soft Tissue Injury	6 (5.4%)	23 (20.5%)	29 (25.9%)

*Patients with both fracture and compartment syndrome were noted as compartment syndrome

** Pelvic fracture included as lower extremity for the table

On the first day after the earthquake, 22 (32.4%) operations were performed, whereas 25 (36.8%) operations were performed on the second day. Fasciotomy was performed on 43 extremities of 32 patients. Of these extremities, 5 (11.6%) were upper and 38 (88.4%) were lower extremities, and the risk of acute compartment syndrome was significantly higher for lower extremities (5/43 vs. 38/43, respectively; *p* = 0.001). One of the upper extremity compartent syndrome was localized in the forearm and 4 of the upper extremity compartment syndrome involved both the arm and forearm. In those diagnosed with lower extremity compartment syndrome, both the thigh and cruris were affected in 4 extremities, and only the cruris was affected in 34 extremities.

**Fig. 3:**
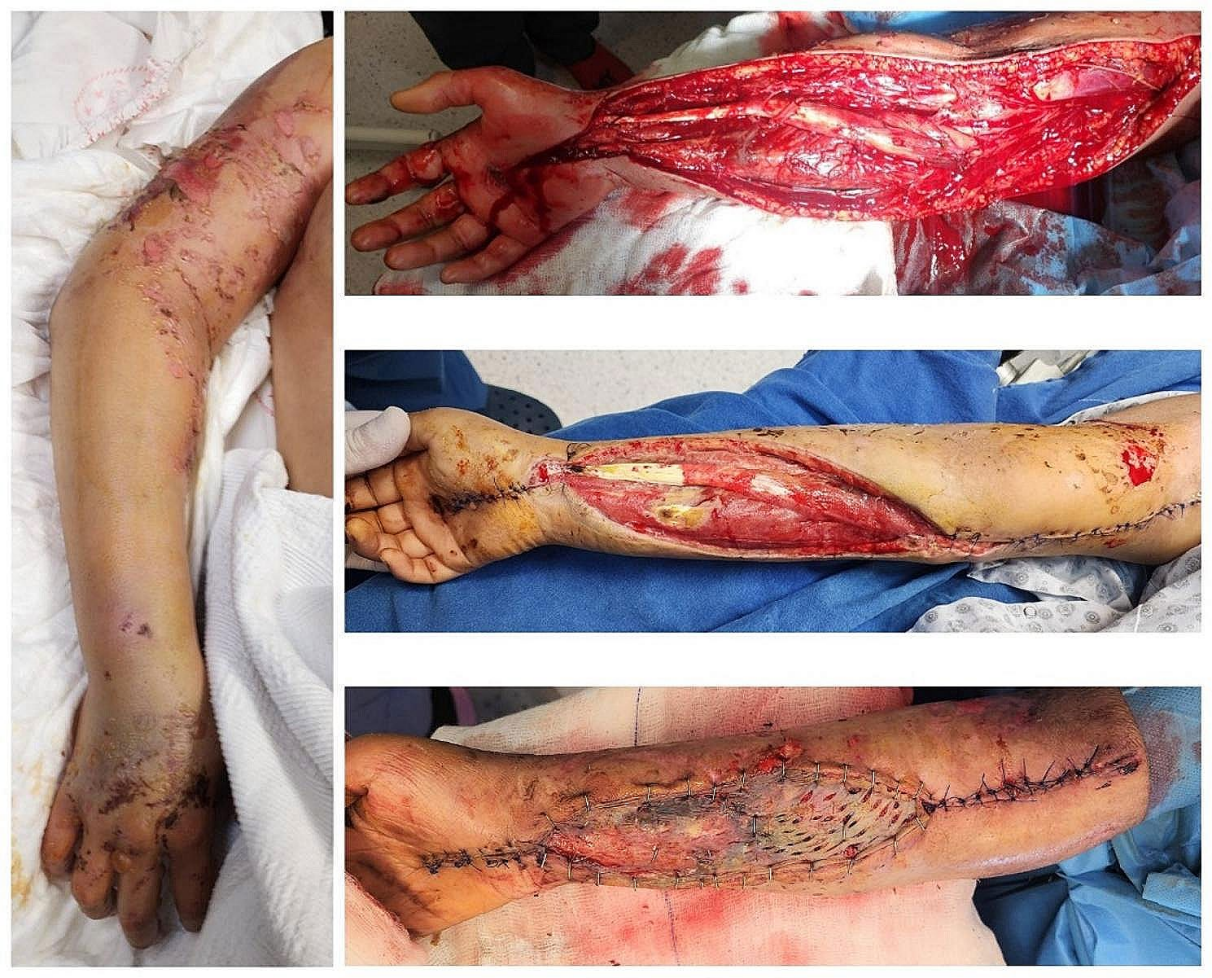
The patient who presented at the 107th hour: (**a**) upper extremity compartment syndrome, (**b**) intraoperative image after emergency fasciotomy completion, (**c**) image after debridment and partial closure, (**d**) after skin grafting

When the patients were divided into two groups based on median age, those < 32 years and those ≥ 32 years old, it was observed that fasciotomy was performed on 20 out of 58 patients (34.4%) < 32 years and on 12 out of 54 patients (22.2%) ≥ 32 years old. No significant difference was found in terms of the acute compartment syndrome rate with regard to age (20/58 vs. 12/54, respectively; *p* = 0.151). The mean time to hospital admission for fasciotomy patients was 34.51 ± 26.13 h (range, 6-107 h) (Fig. 3). Among patients undergoing fasciotomy, 18 (56.2%) were female and 14 (43.8%) were male, and there was no sex-related risk factor for developing acute compartment syndrome (18/32 vs. 14/32, respectively; *p* = 0.858). Out of the 32 patients who underwent fasciotomy, three (9.4%) underwent below-the-knee amputation due to circulatory disorders following the initial surgery, and one of these amputee patients died. All cases of amputation due to compartment syndrome were performed on the lower extremity. Eight (25.0%) of the acute compartment syndrome patients received haemodialysis after fasciotomy. No cases of sepsis were diagnosed after the fasciotomy procedures. No osteomyelitis was observed in any patient who underwent fasciotomy during the 2-month follow-up period. Nevertheless, seven (21.9%) of the patients undergoing fasciotomy died due to crush syndrome (Table 3).

**Table 3:** Comparison of complications between bedside fasciotomy and operating room fasciotomy

Complication	Bedside (*n* = 16)	Operating Theatre (*n* = 16)	P value
Soft Tissue Infection	7 (43.7%)	4 (25.0%)	0.264
Bleeding	2 (12.5%)	1 (6.2%)	0.544
Sepsis	-	-	1.000
Death	3 (18.7%)	4 (25.0%)	0.668
Total	12 (75.0%)	9 (56.2%)	0.456

In three extremities of two patients who had undergone fasciotomy at another centre and were referred to our hospital for follow-up, the previous fasciotomies were insufficient and needed to be expanded. In these two patients, pallor and coldness persisted in the distal extremity, and the capillary refill time was prolonged. One of these patients was a 4-year-old girl, and although widening of the fasciotomy provided improved circulation in the right cruris, below-the-knee amputation was performed for the left cruris during follow-up.

**Fig. 4:**
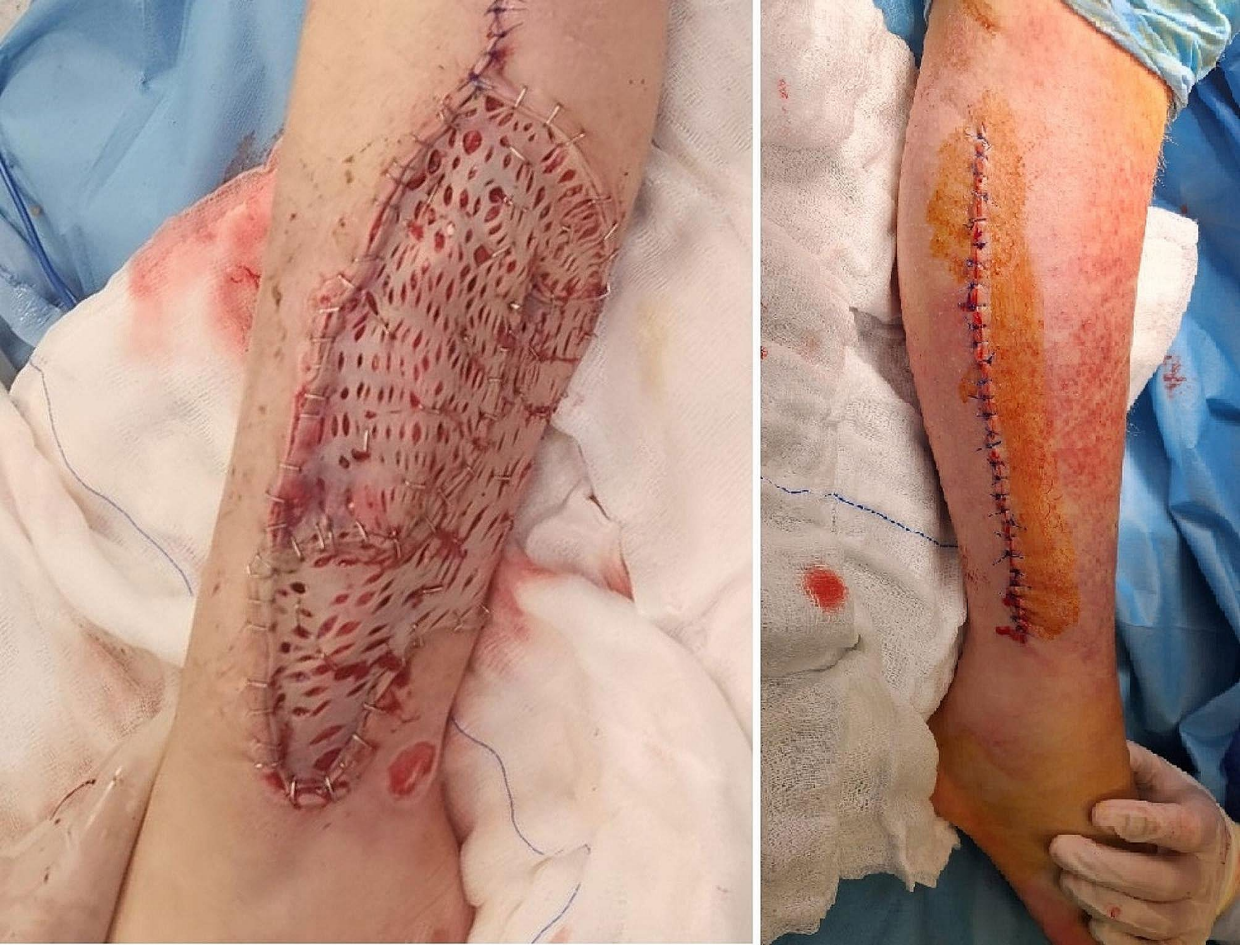
Examples of primary closure and closure with skin grafting. (**a**) Fasciotomy closed with a graft taken from the anterior thigh on the 17th day. (**b**) Primary closure applied on the 12th day of the patient who was in a position to have primary closure after fasciotomy

Primary closure was performed for 19 extremities, whereas 14 extremities were closed with grafting (Fig. 4). The mean time to fasciotomy closure was 11.8 ± 5.66 days (range, 7–25 days).

The surgeries of 16 (50%) of the patients who underwent fasciotomy were performed in the emergency department considering hospital density, operating room availability and patient urgency. One of these patients had upper extremity acs and 15 had lower extremity acs. There was no significant difference in terms of complications or outcomes between performing the fasciotomy at the bedside or in the operating theatre (*p* = 0.456) (Table 3).

Twenty-two (46.8%) of the 47 patients who had fractures were treated surgically, and 25 (53.2%) were treated conservatively (Table 4). The surgery of four patients was postponed due to a shortage of orthopaedic implants.

**Table 4:** Distribution of fracture types and treatment methods

Fracture	N (%)	Treatment	
		*Conservative* *n (%)*	*Surgical* *n (%)*
Clavicle	1 (2.1%)	1 (100%)	-
Proximal Humerus	3 (6.4%)	3 (100%)	-
Humeral Shaft	7 (14.9%)	2 (28.6%)	5 (71.4%)
Olecranon	1 (2.1%)	-	1 (100%)
Forearm Shaft	1 (2.1%)	-	1 (100%)
Forearm Distal	5 (10.6%)	4 (80.0%)	1 (20.0%)
Hand (Phalanx and Metacarpal)	2 (4.3%)	1 (50.0%)	1 (50.0%)
Pelvis	5 (10.6%)	5 (100%)	-
Femoral Shaft	6 (12.8%)	-	6 (100%)
Tibial Eminence	1 (2.1%)	1 (100%)	-
Tibial Shaft	9 (19.1%)	2 (22.2%)	7 (77.8%)
Isolated Fibular Shaft	1 (2.1%)	1 (100%)	-
Calcaneus	2 (4.3%)	2 (100%)	-
Metatarsal	3 (6.4%)	3 (100%)	-
Total	47 (100%)	25 (53.2%)	22 (46.8%)

## Discussion

The current study has presented a cohort of patients with orthopaedic injuries after an earthquake. It was found in this work that fasciotomy represented 47.0% of all operations performed by orthopaedic and trauma surgeons after the earthquake. Fasciotomy appeared to be a crucial surgical procedure for providing health care to the casualties of the earthquake. We have shown that fasciotomy can be safely performed as a bedside procedure based on the urgency of the patient’s condition as well as the availability of the operating theatre. However, the major limitation of the current study was its retrospective, single-institutional design, which might have caused potential selection bias. Absence of complete data in some of these patients is another limitation of the study. In addition, the number of patients was relatively small and statistical methods cannot be used in some data.

The most frequent admissions to hospitals during the first few days following an earthquake are referred to orthopaedics and traumatology [6, 13, 14]. At our hospital, 74.7% of the surgeries performed in the first seven days were carried out by a two-person orthopaedic and traumatology team. The first 36–48 h are the most intense time period at a hospital after an earthquake [15, 16]. In line with the literature, the first two days were the busiest in terms of patient admissions and surgeries at our hospital. Afterwards, the number of patient admissions and surgical procedures decreased. However, we had to postpone four nonurgent surgeries due to a shortage of orthopaedic implants. Although we were unable to find any data on this matter in the literature, there may be shortages in the number of orthopaedic implants available due to sudden increases in trauma cases after an earthquake. Therefore, postponing nonurgent surgeries and planning to use the available resources effectively could be beneficial in the first few days after an earthquake.

Extremity injuries are the most common injuries encountered after an earthquake. In the current study, 42.2% of the patients who visited our hospital had extremity injuries. Several studies revealed that most injuries were associated with the lower extremities [6, 7, 14, 17]. In our study, most of the orthopaedic injuries were located in the lower extremities, in accordance with the literature.

Acute compartment syndrome is a surgical emergency and one of the most common severe conditions diagnosed in causalities after an earthquake [13, 18]. The diagnosis of acute compartment syndrome can be challenging and is based on a combination of clinical findings and pressure measurements within the affected compartment [19]. However, after earthquakes, fasciotomy has generally been carried out without pressure measurements [20]. At our hospital, all acute compartment syndrome diagnoses were made based on clinical findings If there is an opportunity and sufficient time, intracompartmental pressure measurement can be utilized. However, depending on the number of patients in such disasters, diagnosis can be directly determined based on clinical findings. Since clinical findings in compartment syndrome may mask the clinical findings of a concurrent fracture, we recommend that all patients diagnosed with compartment syndrome undergo fracture investigation with plain radiography. Although acute compartment syndrome is more common in males [21, 22], several studies have reported no sex-related difference in the rate of acute compartment syndrome due to crush injuries after an earthquake [23, 24]. Similarly, we did not find a relationship between sex and acute compartment syndrome after earthquake.

The cut-off time for fasciotomy in the treatment of acute compartment syndrome is controversial. However, some studies have reported that muscle damage occurs six hours after the onset of circulatory disorder and that irreversible nerve damage occurs after 12 h [25, 26]. Some authors recommend that fasciotomy should not be performed 24–48 h after trauma since delayed fasciotomy can increase the risk of infection, sepsis, and death [27–29]. However, studies on the outcomes of early and late fasciotomy have shown that the time of fasciotomy has no significant effect on complications such as sepsis and death [30, 31]. We did not impose any time limit on fasciotomy and performed fasciotomy in all acute compartment syndrome patients without findings indicating necrosis. We found that there was a moderate increase in the rate of soft tissue infection in patients who underwent late fasciotomy. However, we observed that late fasciotomy did not have a significant effect on sepsis, crush syndrome, or mortality. The patient who was admitted to our hospital at the 107th hour underwent fasciotomy, which salvaged her upper extremity, and the fasciotomy was closed on the 15th day. However, since we do not have the opportunity to compare functional results, we cannot comment on the functional results of early or late fasciotomy.

Although fractures are known as the most common cause of acute compartment syndrome or the most common accompanying pathology [18, 22], only three (6.9%) of 43 extremities diagnosed with acute compartment syndrome had accompanying fractures in our study. Hope and McQueen [32] showed that patients with acute compartment syndrome without fractures had significantly more muscle necrosis. In the current study, none of the patients with crush syndrome undergoing haemodialysis had any accompanying fractures. Our findings are in line with those reported by Hope and McQueen [32], suggesting that the risk of developing crush syndrome is higher in patients with acute compartment syndrome and no fractures.

Incomplete or inadequate fasciotomy has been related to potentially fatal muscle necrosis [33]. We had two patients who underwent inadequate fasciotomy at other centres, and unfortunately, one of these patients required below-the-knee amputation after we expanded her inadequate fasciotomy. We consider that it may be beneficial to hold meetings for trauma surgeons with updated information on postearthquake interventions, particularly in countries located in earthquake zones.

There have been studies showing that bedside fasciotomy can be applied in emergent conditions, and the outcome of bedside fasciotomy has been reported to be successful [34, 35]. However, to the best of our knowledge, no study has compared the outcomes of bedside fasciotomy and fasciotomy in the operating theatre. In our study, bedside fasciotomy was performed for 16 patients in the emergency department, and there was no significant difference in terms of outcomes or complications compared to fasciotomies performed in the operating theatre (*n* = 16). Thus, under crisis conditions, such as after an earthquake disaster, resulting in a sudden increase in the number of patients, bedside fasciotomy can be performed safely at the time of admission to the emergency department.

## Conclusions

Fasciotomy appears to be a crucial surgical procedure when providing health care for the causalities of an earthquake. Fasciotomy can be safely performed as a bedside procedure based on the urgency of the patient’s condition as well as the availability of the operating theatre. Sharing knowledge and experience about injuries caused by disasters can help to improve preparedness and response plans for future disasters.

## Data Availability

Raw data regarding the dataset is not publicly available in order to protect the privacy of individuals, but can be shared with the editor or referees upon request.

## Declarations

All authors confirm that all methods were carried out in accordance with relevant guidelines and regulations. Institutional committee approval for relevant details and experiments was obtained. Informed consent was obtained from all subjects and/or their legal guardian(s).

## References

[1] USGS. Why are we having so many earthquakes? USGS. https://www.usgs.gov/faqs/why-are-we-having-so-many-earthquakes-has-naturally-occurring-earthquake-activity-been#:~:text=According%20to%20long%2Dterm%20records,earthquake%20magnitude%208.0%20or%20greater.

[2] USGS. M 7.8 - Pazarcik earthquake, Kahramanmaras earthquake sequence. USGS. 2023. https://earthquake.usgs.gov/earthquakes/eventpage/us6000jllz/impact. 2023.

[3] Türkiye Cumhuriyeti Cumhurbaşkanlığı Strateji Ve Bütçe Başkanlığı. 2023 Kahramanmaraş ve Hatay depremleri raporu. Türkiye Cumhuriyeti Cumhurbaşkanlığı Strateji Ve Bütçe Başkanlığı. 2023. https://www.sbb.gov.tr/2023-kahramanmaras-ve-hatay-depremleri-raporu/. Accessed 17 Mar 2023.

[4] Turkish Medical Association. 6 şubat 2023 Kahramanmaraş ve 20 şubat 2023 Hatay depremleri birinci ay raporu. Turkish Medical Association. 2023. https://www.ttb.org.tr/deprem/. 2023.

[5] OCHA. Türkiye: 2023 earthquakes situation report no. 11, as of 23 March 2023 [en/tr]. The United Nations Office for the Coordination of Humanitarian Affairs, OCHA. 2023. https://reliefweb.int/report/turkiye/turkiye-2023-earthquakes-situation-report-no-11-23-march-2023-entr. Accessed 24 Mar 2023.

[6] Zhang L, Zhao M, Fu W, Gao X, Shen J, Zhang Z (2014). Epidemiological analysis of trauma patients following the Lushan earthquake. PLoS ONE.

[7] Mulvey J, Awan S, Qadri A, Maqsood M (2008). Profile of injuries arising from the 2005 Kashmir earthquake: the first 72 h. Injury.

[8] Bartels SA, VanRooyen MJ (2012). Medical complications associated with earthquakes. Lancet.

[9] Hatamizadeh P, Najafi I, Vanholder R, Rashid-Farokhi F, Sanadgol H, Seyrafian S (2006). Epidemiologic aspects of the bam earthquake in Iran: the nephrologic perspective. Am J Kidney Dis.

[10] Duckworth AD, McQueen MM (2017). The diagnosis of acute compartment syndrome: a critical analysis review. JBJS Rev.

[11] Singh K, Bible JE, Mir HR (2015). Single and dual-incision fasciotomy of the lower leg. JBJS Essent Surg Techniques.

[12] Leversedge FJ, Moore TJ, Peterson BC, Seiler JG (2011). Compartment syndrome of the upper extremity. J Hand Surg.

[13] MacKenzie JS, Banskota B, Sirisreetreerux N, Shafiq B, Hasenboehler EA (2017). A review of the epidemiology and treatment of orthopaedic injuries after earthquakes in developing countries. World J Emerg Surg.

[14] Bulut M, Turanoğlu G, Armağan E, Akköse Ş, Özgüç H, Tokyay R (2001). The analysis of traumatized patients who admitted to the Uludağ university medical school hospital after the marmara earthquake. Ulus Travma Derg.

[15] Alkan N, Elmas İ, Karakuş M, Akkay E (2001). Problems encountered during natural disasters: a questionnaire study. Ulus Travma Derg.

[16] Eyler Y, Kılıç TY, Atilla ÖD, Berksoy E. 30 ekim 2020 İzmir depremi sonrası sağlık bilimleri üniversitesi tepecik eğitim ve araştırma hastanesi acil tıp kliniklerine başvuran hastaların analizi. J Tepecik Educ Res Hosp/İzmir Tepecik Eğit ve Araşt Hastan Derg. 2022;32.

[17] Çağıran Z, Sertöz N, Karaman S, Özen D, Demirkoparan M, Uyar M (2023). Our clinical experiences in the earthquake victims who came to our university after the 2020 Aegean Sea earthquake during the COVID-19 pandemic. Ulus Travma Acil Cerrahi Derg.

[18] Via AG, Oliva F, Spoliti M, Maffulli N (2015). Acute compartment syndrome. Muscles Ligaments Tendons J.

[19] Guo J, Yin Y, Jin L, Zhang R, Hou Z, Zhang Y (2019). Acute compartment syndrome: cause, diagnosis, and new viewpoint. Medicine.

[20] Oda J, Tanaka H, Yoshioka T, Iwai A, Yamamura H, Ishikawa K (1997). Analysis of 372 patients with crush syndrome caused by the Hanshin-Awaji earthquake. J Trauma Acute Care Surg.

[21] McQueen M, Gaston P, Court-Brown CM (2000). Acute compartment syndrome: who is at risk?. J Bone Jt Surg Br Vol.

[22] Schmidt AH. Acute compartment syndrome. Evidence-Based Orthop. 2011;627–35. 10.1002/9781444345100.ch71.

[23] Duman H, Kulahci Y, Sengezer M (2003). Fasciotomy in crush injury resulting from prolonged pressure in an earthquake in Turkey. Emerg Med J.

[24] Yang H, Wang J, Kang B, Wang T, Li Y, Wang L (2008). Analysis of the location, early diagnosis and treatment of osteofascial compartment syndrome resulted from Wenchuan earthquake. Zhonghua Wai Ke Za Zhi.

[25] Rorabeck CH, Clarke KM (1978). The pathophysiology of the anterior tibial compartment syndrome: an experimental investigation. J Trauma.

[26] Heckman MM, Whitesides TE, Grewe SR, Judd RL, Miller M, Lawrence JH (1993). Histologic determination of the ischemic threshold of muscle in the canine compartment syndrome model. J Orthop Trauma.

[27] Michaelson M (1992). Crush injury and crush syndrome. World J Surg.

[28] Finkelstein JA, Hunter GA, Hu RW (1996). Lower limb compartment syndrome: course after delayed fasciotomy. J Trauma Acute Care Surg.

[29] Huang KC, Lee TS, Lin YM, Shu KH (2002). Clinical features and outcome of crush syndrome caused by the chi-chi earthquake. J Formos Med Assoc.

[30] Williams AB, Luchette FA, Papaconstantinou HT, Lim E, Hurst JM, Johannigman JA (1997). The effect of early versus late fasciotomy in the management of extremity trauma. Surgery.

[31] Dover M, Memon AR, Marafi H, Kelly G, Quinlan JF (2012). Factors associated with persistent sequelae after fasciotomy for acute compartment syndrome. J Orthop Surg (Hong Kong).

[32] Hope M, McQueen M (2004). Acute compartment syndrome in the absence of fracture. J Orthop Trauma.

[33] Ritenour AE, Dorlac WC, Fang R, Woods T, Jenkins DH, Flaherty SF (2008). Complications after fasciotomy revision and delayed compartment release in combat patients. J Trauma Acute Care Surg.

[34] Ebraheim NA, Abdelgawad AA, Ebraheim MA, Alla SR (2012). Bedside fasciotomy under local anesthesia for acute compartment syndrome: a feasible and reliable procedure in selected cases. J Orthop Traumatol.

[35] Ebraheim NA, Siddiqui S, Raberding C (2016). A single-incision fasciotomy for compartment syndrome of the lower leg. J Orthop Trauma.

